# Performance Analysis of Full Assembly Glass Fiber-Reinforced Polymer Composite Cross-Arm in Transmission Tower

**DOI:** 10.3390/polym14081563

**Published:** 2022-04-11

**Authors:** Agusril Syamsir, Afiqah Nadhirah, Daud Mohamad, Salmia Beddu, Muhammad Rizal Muhammad Asyraf, Zarina Itam, Vivi Anggraini

**Affiliations:** 1 Institute of Energy Infrastructure, Universiti Tenaga Nasional, Jalan IKRAM-UNITEN, Kajang 43000, Selangor, Malaysia; 2 Department of Civil Engineering, Universiti Tenaga Nasional, Kajang 43000, Selangor, Malaysia; fiqanadhirah@gmail.com (A.N.); Daud@uniten.edu.my (D.M.); Salmia@uniten.edu.my (S.B.); iZarina@uniten.edu.my (Z.I.); 3 Civil Engineering Discipline, School of Engineering, Monash University Malaysia, Jalan Lagoon Selatan, Bandar Sun Way, Subang Jaya 47500, Selangor, Malaysia; vivi.anggraini@monash.edu

**Keywords:** GFRP, pultruded composite, cross-arm, transmission tower, finite element analysis

## Abstract

The usage of glass fiber reinforced polymer (GFRP) composite cross-arms in transmission towers is relatively new compared to wood timber cross-arms. In this case, many research works conducted experiments on composite cross-arms, either in coupon or full-scale size. However, none performed finite element (FE) analyses on full-scale composite cross-arms under actual working load and broken wire conditions. Thus, this work evaluates the performance of glass fiber reinforced polymer (GFRP) composite cross-arm tubes in 275 kV transmission towers using FE analysis. In this study, the performance analysis was run mimicking actual normal and broken wire conditions with five and three times more than working loads (WL). The full-scale assembly load test experiment outcomes were used to validate the FE analysis. Furthermore, the mechanical properties values of the GFRP composite were incorporated in simulation analysis based on the previous experimental work on coupons samples of GFRP tubes. Additionally, parametric studies were performed to determine the ultimate applied load and factor of safety for both normal and broken wire loading conditions. This research discovered that the GFRP composite cross-arm could withstand the applied load of five times and three times working load (WL) for normal and broken wire conditions, respectively. In addition, the factor of safety of tubes was 1.08 and 1.1 for normal and broken wire conditions, respectively, which can be considered safe to use. Hence, the composite cross-arms can sustain load two times more than the design requirement, which is two times the working load for normal conditions. In future studies, it is recommended to analyze the fatigue properties of the composite due to wind loading, which may induce failure in long-term service.

## 1. Introduction

Transmission towers are electrical pylons that function to support overhead power lines. The transmission tower designs were categorized into two types, namely, monopole steel tubes and latticed steel towers. The transmission tower lines usually supply electrical power from the power generator to sub-stations before going to consumers [1]. The power cables on the transmission tower carry high power voltage to a typical height of 15 to 55 m above the ground [2,3,4,5]. Generally, the latticed steel towers were installed across Peninsular Malaysian states since 1929 [6,7,8]. The latticed tower comprises several components such as a cross-arm, peak, boom, tower body and cage. In this manner, the cross-arm component plays a vital role in grasping and securing the power cables with its insulators above the ground.

Generally, the materials used for cross-arms in transmission towers are wood, steel and Glass Fiber Reinforced Polymers (GFRP) [9,10]. Historically, wooden timber from Chengal wood (*Neobalanocarpus hemii*) is used due to its excellent strength and arc quenching during the strike of lightning. However, the wooden cross-arms started to experience failure after giving the service for more than 20 years [11]. The failure of wood-based material occurred due to natural wood defects [12,13,14], microbial and biological attack [15,16,17,18] and creep [19,20]. Additionally, the availability of good quality wood timbers for structure has declined every year and has expedited researchers to find alternatives for wood cross-arms [21,22,23]. Wood timber was later replaced with steel due to its flexible design and lightweight material. However, despite having advantages, steel is easily corroded due to the environment, chemicals and pollution. In addition, steel is also electrically conductive, which will endanger installing personnel. From this point of view, fiber reinforced polymer (FRP) composites such as GFRP were proposed as replacements for wood timber and steel cross-arms due to their lightweight, high mechanical performance as well as good thermal and electrical insulation properties to ensure their sustainability in supplying electrical power to consumers [24,25,26,27]. In general, FRP composites are widely used in many sectors, including high durability and military applications [28,29,30], construction and building material [31,32,33,34], household and office appliances [35,36,37], medical tools and instruments [38,39], aviation components [40,41,42] and automotive parts [43,44,45]. Due to the outstanding properties of the FRP composites, a kick-starter pilot project on a GFRP composite cross-arm in a 132 kV transmission tower was commenced in the Tanjung Batu line within Pekan Town, Pahang, Malaysia, by Abu Bakar et al. [46].

Recently, several kinds of research were carried out involving the various modes of studies in order to attain technical data on the influence of material configurations on the cross-arm’s integrity. In this case, Asyraf et al. [47] developed a full-scale test rig specialized for cross-arm assembly to evaluate their physical and structural performance. After test rig development, several mechanical tests were executed to evaluate the static and creep properties of wooden [20,48] and GFRP composite cross-arms [49]. In the earlier stage, many researchers were working on characterizing coupon specimens of cross-arms in long-term services, such as Johari et al. [14,15] and Asyraf et al. [19]. Most of these studies were performed using a coupon-scale creep test rig developed by Asyraf et al. [50]. Table 1 display the recent progress of GFRP composite cross-arm studies conducted by various researchers.

Even though many studies were implemented experimentally, it can be found that there is a lack in the analysis of computer simulation for GFRP cross-arms. Most of the literature revealed that the existing design of the GFRP cross-arm is still impended due to failure that occurred on the GFRP cross-arm after six months of installation [53,54,55]. From this point of view, the GFRP composite cross-arm has limitations, especially in design codes availability [56]. In order to solve this issue, a computer simulation analysis has to be applied to investigate the structural performance of GFRP composite cross-arms during service. This approach will contribute to the comprehension and forecasting of the mechanical durability of existing composite structures, providing a holistic and intuitive perspective for investigating the structure [56,57]. In recent decades, costs related to the construction and maintenance of the transmission tower’s cross-arm have become crucial because they will affect electricity production.

This research article is expected to demonstrate the parametric studies conducted on the GFRP composite cross-arm in a 275 kV transmission tower using numerical modelling to measure its ultimate load and factor of safety. At the end of the study, the research outputs from this analysis are intended to set a baseline for mechanical profiling of full-scale GFRP composite cross-arms. Thus, the outcomes of this study are projected to provide a practical perspective to researchers and engineers for understanding the mechanical performance of GFRP composite cross-arms.

## 2. Methodology

**Figure 1:**
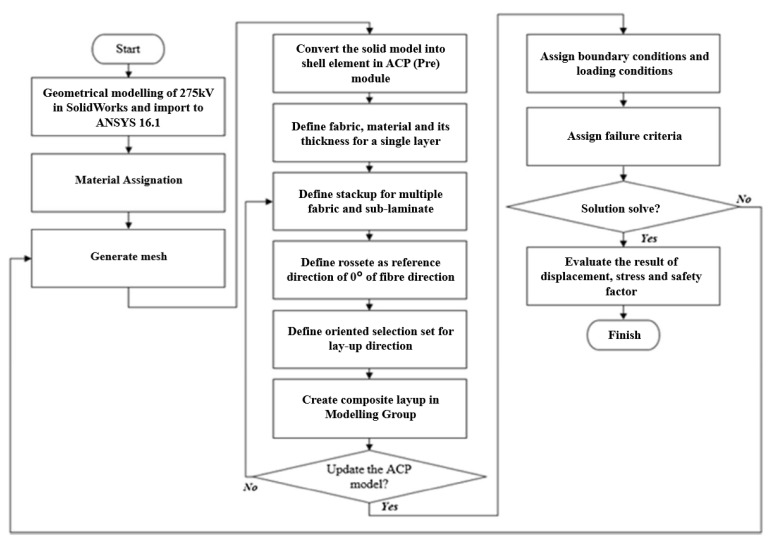
Workflow of computational simulation analysis of cross-arm assembly.

In this study, the structural analysis was commenced using the modelling process of the finite element (FE) simulation, replicating the real conditions of the experiment. The initial inputs such as mechanical properties of GFRP composite cross-arm were installed in the FE simulation after finalizing the size and dimension GFRP composite cross-arm model. These inputs of the GFRP composite are density (2.03 g/cm^3^), Poisson ratio (0.28) and Young’s modulus in the x-axis (34,000 MPa), y-axis (10,200 MPa) and z-axis (3100 MPa), and they were added to the structural analysis. The inputs were updated in the setting of the computational simulation correlating the fundamental of the laminated composite laminate modelling. The overall process is displayed in Figure 1.

### 2.1. Geometrical Configurations

**Figure 2:**
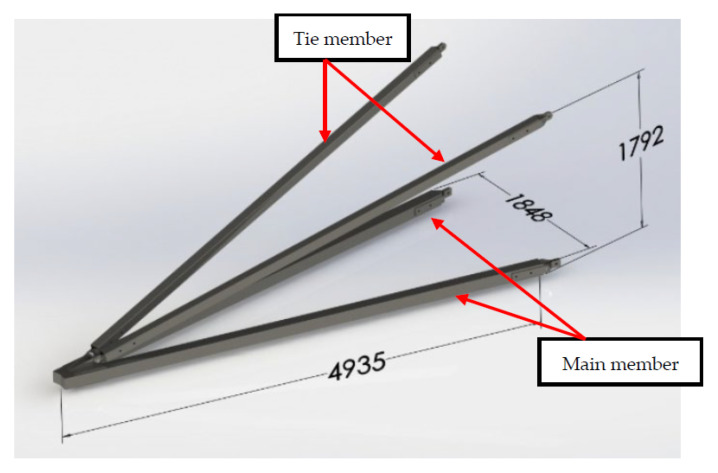
Three-dimensional view and dimension of GFRP composite cross-arm (in mm).

In this work, the cross-arm assembly was designed using SolidWorks. Thus, it would produce a more accurate drawing configuration of the structure. The primary structural component of cross-arms comprises four members, such as two main members and two tie members. The general height, length and width of cross-arm assembly is 1792 mm, 4935 mm and 1848 mm. The design of a 24 L GFRP composite cross-arm in a 275 kV transmission tower is depicted in Figure 2.

**Figure 3:**
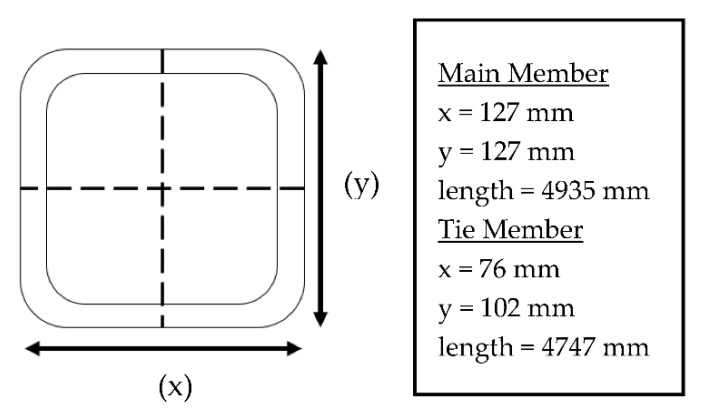
Schematic diagram of cross-section dimension of GFRP cross-arm for main and tie members.

A schematic diagram of the cross-sectional dimensions of the GFRP tube can be observed in Figure 3. The thickness of the cross-section was relatively constant, between 6.3 to 6.5 mm. The fiber content and densities were relatively constant on all sides and even in the corners. The average fiber volume fraction recorded was 70.5%. Generally, the GFRP cross-arm in the 275 kV transmission tower is made up of nine layers of glass fibers, of which five layers (laminas) of continuous fiber rovings are interspaced with four layers (laminas) of the stitched glass fiber mat. This lamination sequence of the GFRP structure is based on the findings reported by Muttashar et al. [58] on Brand X pultruded samples.

### 2.2. Material Assignation

According to previous studies, the GFRP composite cross-arm comprises pultruded glass fiber reinforced unsaturated polyester composites [59,60]. The glass fiber composite was chosen as the cross-arms building material for its excellent bending strength, high tensile properties and remarkable thermal and electrical insulation properties [61,62]. The GFRP composite is commonly fabricated from E-glass fiber reinforced with unsaturated polyester (UPE) resin with a ratio of 37:63 in order to achieve optimum performance using the pultrusion process [63,64]. Pultrusion facilitates the fabrication process of cross-arm beams by impregnating the fiber with a thermosetting matrix and is pulled via a heated die. The surface finish of composite cross-arm beams is homogenous and fine surface laminate. In some cases, the composite beams also are embedded with calcium carbonate filler to improve the water resistance of the composite structure [65]. Generally, the GFRP composite forms a lightweight material since both E-glass fiber and UPE exhibit low densities, 2580 and 1350 kg/m^3^, respectively.

Experimental testing was performed on a 275 kV 24 L type GFRP cross-arm. Many manufacturers produce different brands of GFRP cross-arm. One of the brands considered in this study is the GFRP composite cross-arm. The engineering data and material properties collected from mechanical testing are required as input parameters in numerical modelling. Therefore, specimens were cut from a different side of the GFRP cross-arm and were tested according to ASTM standards presented in ASTM D792, ASTM D3039 and ASTM D695. The results are presented in Table 2.

### 2.3. Mesh Generation

Meshing is a critical element in finite element analysis to determine the accuracy of validating the node generated and define the output of the analysis. Before generating the mesh for the design configuration, it is essential to confirm that it should represent the computational domain and loading. Kanesan et al. [66] stated that the mesh has to optimally represent the solution’s large-displacement or stress gradient. In this work, the shell element was applied to analyze composite shells/tubes. For a laminated shell, the orientation of each lamina is defined as a rotation angle. The orientation of each lamina is relative to the orientation of the entire shell section. This study implemented mesh with the accurate solution for hexahedral mesh.

### 2.4. Experimental Program

**Figure 4:**
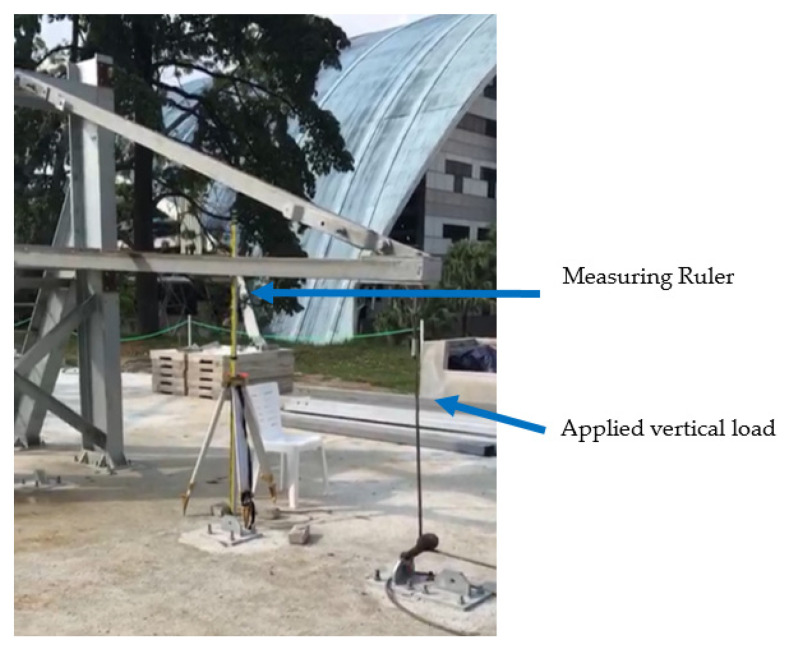
Full Assembly testing of GFRP cross-arm subjected to vertical load.

Complete assembly testing of the GFRP cross-arm was conducted to determine the maximum deformation at the middle of the main member and the failure load. Full-scale testing was set up as shown in Figure 4. Vertical loading started when 21,248 N load was applied until the cross-arm failed to withstand its structure.

The deformation was measured at 30 min using a ruler tied to the tripod and placed in the middle of the cross-arm. Deformation at 30 min and the breaking load was recorded and compared with numerical modelling results. Deformation at 30 min and breaking load were 77 mm and 80 kN, respectively.

### 2.5. FE Analysis

#### 2.5.1. Finite Element Model and Validation

Numerical modelling of the GFRP cross-arm was developed using ANSYS software. ANSYS Composite Prepost (ACP) was used as a sub-component system to analyze the layer of the GFRP cross-arm. SOLID46 elements were suitable to mesh the GFRP cross-arm model [67]. The material properties tabulated in Table 2 was used as the input parameters. An eight-node with three degrees of freedom x, y and z was input to develop these numerical models.

**Figure 5:**
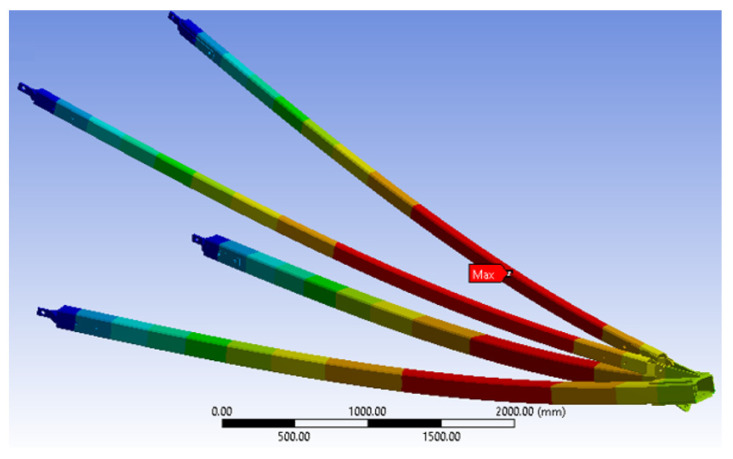
Typical deformed shape of GFRP cross-arm.

Numerical analysis was performed to attain the deformation shape and values of the GFRP structure in vertical loading, as shown in Figure 5. The FE model was later verified with the experimental work and it was discovered that the GFRP cross-arm’s deformation result from the experimental work was 77 mm, while the static structural analysis result shows the deformation was 76.8 mm. Thus, the percentage difference is only 0.23%, which shows that the FE analysis accurately presents the actual loading conditions. According to Tian et al. [68] and Sayahi et al. [69], the acceptable range of percentage error is 20–25%. Generally, the percentage errors to validate numerical results are divided into five classes, which are highly acceptable (0.1–9.9% accuracy), good (10–14.9% accuracy), satisfactory (15–19.9% accuracy), fair (20–24.9% accuracy) and unsatisfactory (more than 25 accuracy value) [70]. These findings showed that numerical results forecast the deformation values with highly acceptable accuracy. Therefore, this study established that the FE analysis discovered to elaborate the deformation value of the GFRP cross-arms was verified with precise and consistent values from the experimental outcome. Figure 5 show the typical deformed shape of the GFRP cross-arm when the vertical load was applied.

#### 2.5.2. Parametric Studies

In this section, a parametric study was conducted using numerical modelling involving two cases: a normal condition and a broken wire condition. The condition, when all the wires are intact at the GFRP cross-arm, is called a normal condition, while the condition when the simulation conductor or earth wire is discovered broken is called the broken wire condition. The variable parameters of these parametric studies are load and deformation. Other than that, the failure of the GFRP cross-arm was also calculated by the minimum Factor of Safety (FOS) [71]:

(1) $$\frac{\mathrm{Compressive}\;\mathrm{Strength}\;\left({N/\mathrm{mm}}^{2}\right)}{(\mathrm{Stress}\;\mathrm{Ply}\;\mathrm{of}\;\mathrm{GFRP}\;\mathrm{First}\;\mathrm{Layer}\;\left({N/\mathrm{mm}}^{2}\right)}\;>\;1.0$$

**Figure 6:**
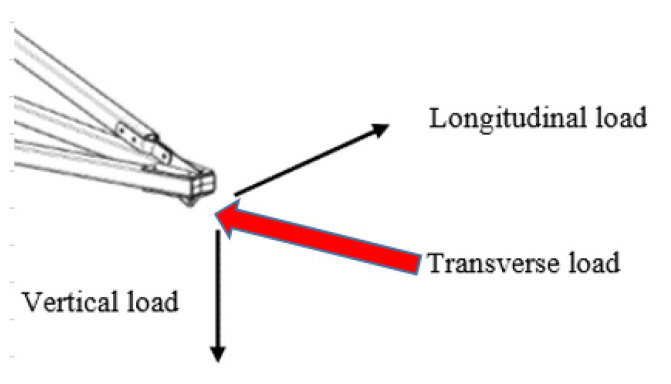
Working load applied in GFRP cross-arm.

Non-linear analysis was carried out to obtain the maximum working load (WL), deformation and minimum safety factor. The working load shown in Figure 6 was applied on the GFRP cross-arm due to wire loading, which creates three loads: longitudinal, vertical and transverse [48,72]. The load applied to the cross-arm is shown in Table 3 as a vertical and transverse load only, while for broken wire conditions, these three loads act together with the cross-arm.

## 3. Results and Discussions

### 3.1. Analysis for Normal Condition

**Figure 7:**
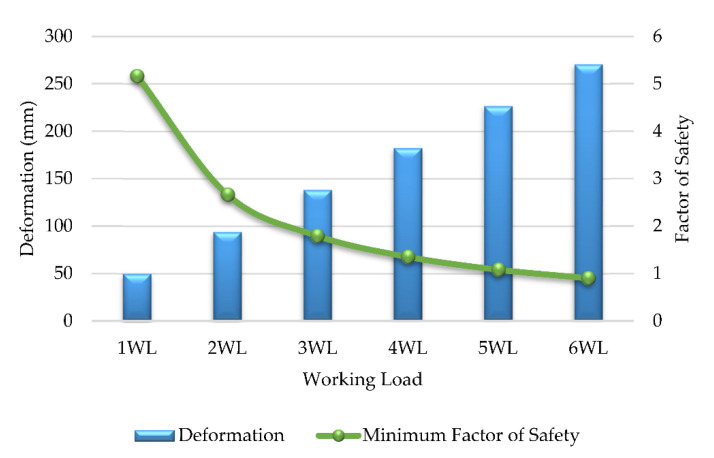
Deformation and Factor of Safety (FOS) for GFRP cross-arm (Normal condition).

As discussed in this section, the deformation, stress ply, compressive strength and safety factors of the GFRP cross-arm were implemented for normal conditions, as shown in Table 4. Figure 7 display that the maximum working load the GFRP cross-arm can withstand is 5WL before the failure. The maximum working load held by the GFRP cross-arm is 106,240 N and 58,590 N for vertical and transverse loads, respectively. The maximum deformation of composite cross-arm right before it fails is 226.35 mm. In addition, the minimum Factor of Safety (FOS) is 1.08, which is higher than 1, which is considered safe. The maximum stress ply for the first layer of the main member of the GFRP cross-arm is 296.12 N/mm^2^, while the maximum stress ply for the first layer of the tie member is 275.28 N/mm^2^.

Table 4 display that the layering of E-glass fiber with unsaturated polyester exhibits good stress ply value at the first layer up until 5 WL. This phenomenon occurred due to the delayed breakage of glass fiber as the stress was applied as glass fiber exhibits high tensile strength and strength-to-weight ratio [73]. The delayed breakage of glass fiber occurred as the molecular chain of UPE resin more prospectively slipped and stretched during a high elastic rate. Additionally, good adhesion bonding between E-glass fiber and UPE resin allows better even stress transfer between the fiber layer and the matrix [50]. From all the mentioned points above, it can be seen that the FOS of the GFRP cross-arm is within the safety limit up until 5 WL, where the safety value is more than 1. Therefore, based on Equation (1), the FOS of the GFRP cross-arm must be higher than 1 to be consider safe.

**Figure 8:**
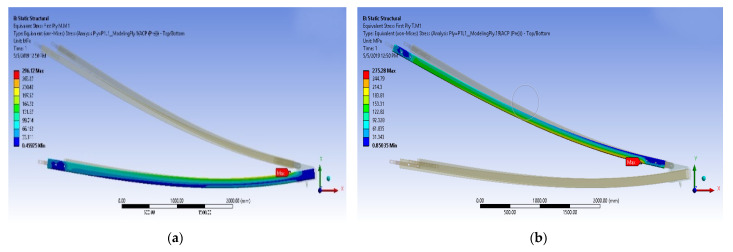
(**a**) Equivalent stress ply first layer of the main member (**b**) Equivalent stress ply first layer of tie member.

Figure 8a,b show the maximum deformation; equivalent stress first ply for the main member and tie member of composite cross-arms for 5 × Working Load (WL). Bending of the composite cross-arm toward the middle member can be found. Additionally, it can also be observed that maximum stress for both main and tie members is located near to vertical load, which may initiate the crack propagation later on. Besides that, the equivalent stress ply first layer stress of the main member is higher than the tie member due to the tie member acting as a support in the cross-arm structure.

### 3.2. Analysis for Broken Wire Condition

**Figure 9:**
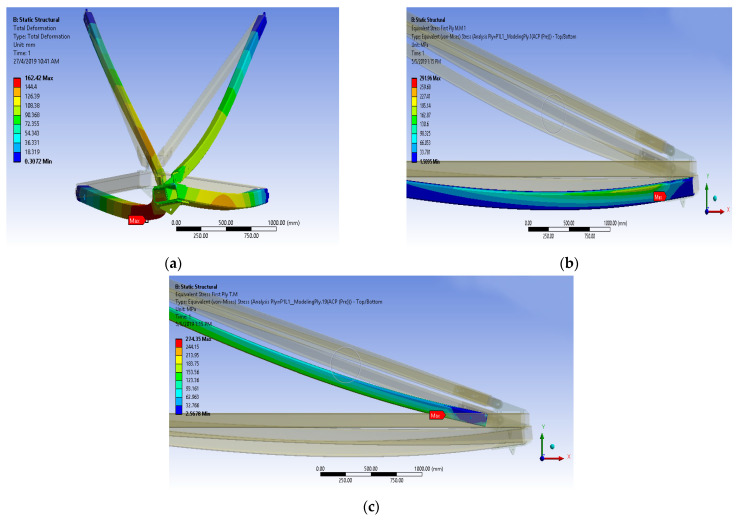
(**a**) Maximum deformation of GFRP cross-arm in broken wire condition (**b**) Equivalent stress ply first layer of the main member (**c**) Equivalent stress ply first layer of tie member.

Results of parametric study for GFRP composite cross-arm in broken wire condition are shown in Table 5. From the outcome, the maximum working load that the GFRP cross-arm can withstand for broken wire conditions is 3WL before the failure. The maximum load held by the composite cross-arm for broken wire condition is 49,308 N, 26,001 N and 77,337 N for vertical, transverse, and longitudinal load, respectively. Figure 9 show the GFRP composite cross-arm FE simulation under broken wire conditions. From this point of view, it can be concluded that the broken wire condition permits the cross-arm to withstand lesser load magnitude. This finding is due to the twisting effect that induces a torsional irregularity reaction for the whole cross-arm assembly, which causes the structure to fail after loading 3 WL. According to Gokdemir et al. [66], the structure without bracing with a separation distance would increase the lateral rigidity in the weak direction of the structure. This condition would result in structural failure due to torsion actions.

**Figure 10:**
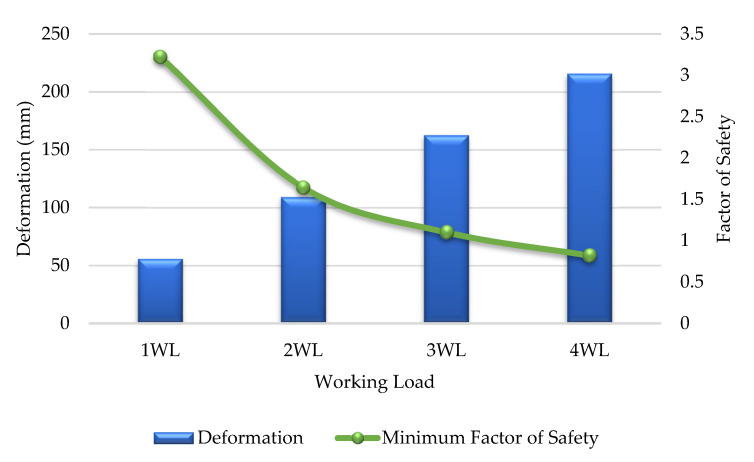
Deformation and Factor of Safety (FOS) for GFRP cross-arm (broken wire condition).

In conjunction with this issue, the Factor of Safety (FOS) is 3.22, higher than 1, which is considered safe. Referring to Table 5, the maximum deformation of the GFRP composite cross-arm right before it fails is 162.42 mm at a FOS of 1.10 for broken wire conditions. The maximum stress ply for the first layer of the main member of the GFRP cross-arm is 291.96 N/mm^2^, while the maximum stress ply for the first layer of the tie member is 274.35 N/mm^2^. Figure 10 display the trends of deformation and safety factor for the GFRP composite cross-arm versus the loading concentration applied in broken wire conditions.

### 3.3. Factor of Safety

**Figure 11:**
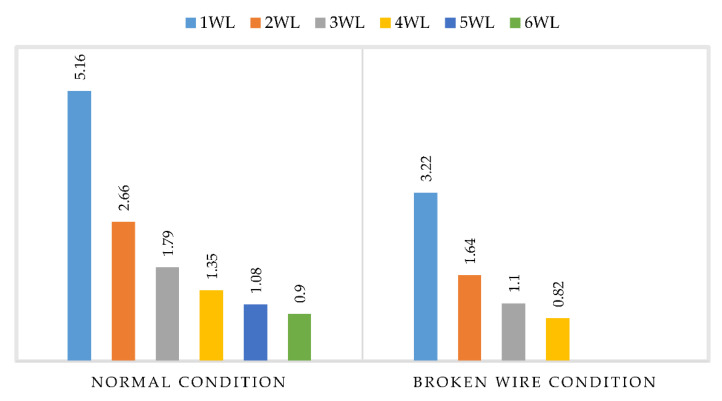
Comparison Factor of Safety (FOS) for normal and broken wire condition.

In this section, the factor of safety on both conditions, normal condition and broken wire condition, was studied. The summarized safety factor for the GFRP composite cross-arm is presented in Table 6. Table 6 show that the minimum FOS for the normal condition is 1.08 before its failure. Meanwhile, the minimum FOS for the broken wire condition recorded 1.10 FOS before it failed at 0.82 FOS. Figure 11 present the comparison FOS for both conditions, the GFRP composite cross-arm conditions.

In conjunction with these findings, it can be deduced that the maximum loading for normal and broken wire conditions was only withstood up to five and three times higher than WL. This finding proved that the composite cross-arm can withstand any fiber tensile and compressive damages below these values. Higher values than this result in compressive fiber damage, with the highest displacement taking place in the localized around the area of the pinned arms. Subsequently, it would cause composite damage initiation, which leads to structural failure. This study is aligned with the findings led by Daud et al. [74].

### 3.4. Failure Criteria (Hashin Theory)

Failure criteria is a parameter that evaluates various failure modes when more than one stress is applied. For Hashin criteria, it is a parametric analysis that indicates the failure indication comprised of fiber and matrix failures with both tension and compression. Failure of the cross-arm was measured using the Hashin theory analyzed in ANSYS software. Five layers (laminas) of continuous fiber rovings were interspaced with four layers (laminas) of the stitched glass fiber mat. The strength ratio was evaluated to check which lamina (layer) would fail first when the load was applied. The lamina of composite were considered to fail if the value was equal or more than 1.0, as calculated in Equations (2)–(5). Fiber tension and compression are calculated in Equations (2) and (3). Fiber matrix tension and compression are calculated in Equations (4) and (5).

(2) $$\begin{array}{cc} \text{Fiber Tension}\; & {\left(\frac{\sigma_{11}}{X_{T}}\right)}^{2}+\frac{\sigma_{12}^{2}+\sigma_{13}^{2}}{S_{12}^{2}}=\{\begin{array}{c} \geq 1\;\mathrm{failure}\;\\ <1\;\mathrm{no}\;\mathrm{failure} \end{array} \end{array}$$

(3) $$\begin{array}{cc} \text{Fiber Compression}\; & {\left(\frac{\sigma_{11}}{X_{C}}\right)}^{2}=\{\begin{array}{c} \geq 1\;\mathrm{failure}\;\\ <1\;\mathrm{no}\;\mathrm{failure} \end{array} \end{array}$$

(4) $$\text{Matrix Tension}\;\frac{{\left(\sigma_{22}+\sigma_{33}\right)}^{2}}{Y_{T}^{2}}+\frac{\sigma_{23}^{2}-\sigma_{22}\sigma_{33}}{S_{23}^{2}}+\frac{\sigma_{12}^{2}+\sigma_{{}_{13}}^{2}}{S_{12}^{2}}=\{\begin{array}{c} \geq 1\;\mathrm{failure}\;\\ <1\;\mathrm{no}\;\mathrm{failure} \end{array}$$

(5) $$\text{Matrix Compression}\;\left[{\left(\frac{Y_{C}}{2S_{23}}\right)}^{2}-1\right]\left(\frac{\sigma_{22}+\sigma_{33}}{Y_{C}}\right)+\frac{{\left(\sigma_{22}+\sigma_{33}\right)}^{2}}{4S_{23}^{2}}+\frac{\sigma_{23}^{2}-\sigma_{22}\sigma_{33}}{S_{23}^{2}}\;+\frac{\sigma_{12}^{2}+\sigma_{13}^{2}}{S_{12}^{2}}=\{\begin{array}{c} \geq 1{}^{\circ}\;\mathrm{failure}\;\\ <1{}^{\circ}\;\mathrm{no}\;\mathrm{failure} \end{array}$$

where

*σ* = Stress in plane

*X_t_* = Longitudinal tensile strength

*X_c_* = Longitudinal compressive strength

*Y_t_* = Tensile strength in transverse direction

*Y_c_* = Compression strength in transverse direction

*S*_12_ = Longitudinal shear strength

*S*_23_ = Transverse shear strength

The results of the strength ratio for the GFRP composite cross-arm for Hashin theory are shown in Table 7. Laminate represents the stacking of several layers of fiber reinforcement and matrix. Lamina represents the layer of fiber with different orientations. The lamination of the GFRP cross-arm in the 275 kV transmission tower had nine layers, and the sequence was 0/45/0/−45/0/−45/0/45/0. The plot of strength ratio versus different loads for the GFRP composite cross-arm is plotted in Figure 11.

**Figure 12:**
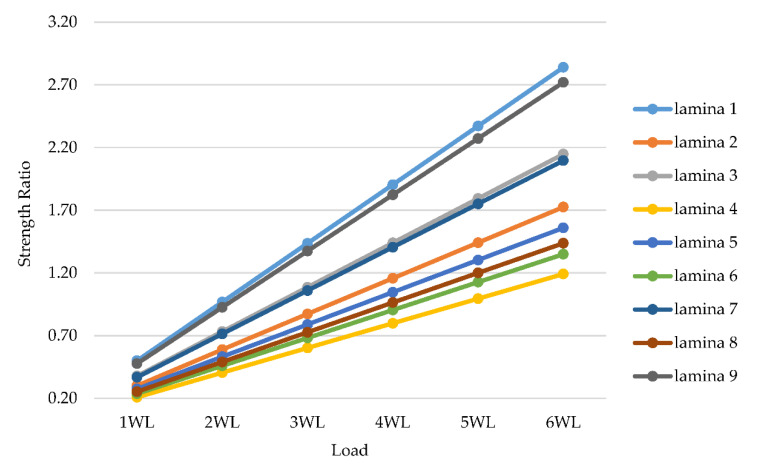
Strength ratio with different loads for GFRP composite cross-arm.

Based on Figure 12, it was discovered that lamina 1, 3, 7 and 9 fail first at three times Working Load (WL) with the values of 1.44, 1.09, 1.06 and 1.37 and 0° fiber orientation. GFRP composite cross-arm has internal failure since most internal lamina failed first for at least 3 WL, but the structure did not fail up until 5 WL. It was observed that the lamina failed due to the deflection at 0° fiber orientation. The failure continued to the other degrees of fiber orientation which were 45° and 90° fiber orientation, due to the torsional effect of failure. Meanwhile, lamina 2 and lamina 5 failed at four times Working Load (WL) with 1.16 and 1.05 strength ratio and 45° and 0° fiber orientation. At five times Working Load (WL), lamina 6 and lamina 8 failed with the values of 1.13 and 1.20, respectively. The last lamina that failed was lamina 4 at six times Working Load (WL) and −45° fiber orientation. From this point of view, Mei et al. [75], Riccio et al. [76] and Nurazzi et al. [77] confirmed that the composite plate with 45° oriented plies would exhibit higher resistance toward impact intensity as compared to 0°/90° ply configuration.

## 4. Conclusions

The effect of load magnitudes and conditions on structural properties of GFRP composite cross-arm assembly was evaluated in this FE study. The analytic forecasting analysis displayed that the stress state developed based on the variation of load magnitude and loading conditions of the structure and remarkably affected the strength. The simulated work was validated with the experiment from the FE analysis. The difference between experimental and numerical deformation shows a difference of 0.02 cm (0.23%), which falls below 5%. The allowable percentage difference between experimental and numerical analysis must fall below 5% to be considered acceptable. Two different loading conditions, such as normal and broken wire conditions, are simulated with six load levels. In this case, the six load levels were set based on between one to six times working loads (WL) for both conditions, in which the maximum load capacity of the cross-arm was 5 and three times WL for normal and broken wire conditions, respectively. The simulated results showed that the GRFP cross-arm’s minimum safety factor was 1.08 and 1.1 for normal and broken wire conditions, respectively. These results showed that the GFRP composite cross-arm’s current design is susceptible to resisting the heavy loading from power cables and insulators in both normal and broken wire conditions with good safety values. Additionally, the composite cross-arm can withstand up to five times WL before its lamina fails with −45° orientation. Therefore, it can be concluded that the current cross-arm can sustain load two times as compared to the design requirement, which is two times the working load for normal conditions.

Future studies recommend evaluating the influence of joint connections on the creep properties of the GFRP cross-arm in a 275 kV transmission tower. Moreover, it is suggested that the current composite cross-arm may be subjected to dynamic loading due to the wind effect over the long-term period. The dynamic loads from the wind would cause the structure to be fatigued and later fail at a certain point in time. The study on the fatigue resistance and strength of GFRP composite cross-arms extends into another dimension of analysis. Thus, this study would broaden the information and perspective in full-scale cross-arm research for both engineers and researchers.

## Figures and Tables

**Table 1:** Current research progresses of GFRP composite cross-arm studies.

Mode of Study	Research	Findings	Ref
Mechanical test rigs development specialized for cross-arms	Conceptual design of creep testing rig for full-scale cross-arm.	- The study implements the TRIZ inventive principles to identify actual test rig problems, morphological chart methods to refine design features and analytic network processes used to select designs. Concept designs five and three were chosen for full-scale and coupon-scale cross-arm test rigs.	[2,47]
	Conceptual design of multi-operation outdoor flexural creep test rig		[50]
Design of GFRP cross-arms	Conceptual design of a braced composite cross-arm	- This research covered developing an optimized bracing design for cross-arm assembly in a 132 kV transmission tower. In addition, this study implements hybridizing of the TRIZ-morphological chart–ANP technique to develop an optimized design. In the end, concept design two was chosen as the optimal design to be used in the cross-arm structure.	[51]
Experiments	Experimental testing on the compressive strength equation for GFRP square tube columns	- Short and intermediate PGFRP beam-columns exhibited a significant reduction of capacity due to the interaction of rushing, local buckling and global buckling, which correspond to each failure.	[52]
	Mechanical evaluation on composite cross-arm performance	- The axial forces in the main member beams are linearly varying with applied load, whereby the tie member of cross-arms that experience axial forces is lesser in magnitude.	[4]

**Table 2:** Mechanical properties of GFRP composite cross-arm tube.

Parameter	Value
Density	2.03 g/cm^3^
Young’s modulus in *x*, *E_x_*	34,000 MPa
Young’s modulus *y*, *E_y_*	10,200 MPa
Young’s modulus *z*, *E_z_*	3100 MPa
Poisson’s ratio (*v_xy_* = *v_yz_* = *v_xz_*)	0.28
Shear modulus (*G_xy_* = *G_yz_* = *G_xz_*)	4280 MPa
Ultimate tensile stress, *σ_t,x_*	429 MPa
Ultimate compressive stress, *σ_c,x_*	320 MPa
Ultimate tensile stress, *σ_t,y_*	100 MPa
Ultimate compressive stress, *σ_c,y_*	76 MPa
Ultimate shear stress, *S_xy_*	95 MPa
Ultimate shear stress, (*S_xz_ = S_yz_*)	70 MPa

**Table 3:** Working load (WL) for 24 L GFRP cross-arm in 275 kV transmission tower for normal and broken wire condition.

	Normal Condition (All Wires Intact), (N)	Broken Wire Condition, (N)
Vertical	21,248	16,436
Transverse	11,718	8667
Longitudinal	0	25,779

**Table 4:** Deformation, stress ply, compressive strength and factor of safety of GFRP cross-arm for normal condition.

Load Designation	Normal Loads (N)		Deformation (mm)	Stress ply (N/mm^2^) First Layer		Compressive Strength (N/mm^2^)	Minimum Factor of Safety (Strength /Stress)
	Vertical	Transverse		Main	Tie		
1 WL	21,248	11,718	49.60	61.98	56.89	320	5.16
2 WL	42,496	23,436	93.77	120.52	111.49	320	2.66
3 WL	63,744	35,154	137.96	179.05	166.09	320	1.79
4 WL	84,992	46,872	182.15	237.59	220.69	320	1.35
5 WL	106,240	58,590	226.35	296.12	275.28	320	1.08
6 WL	127,488	70,308	270.54	354.66	329.88	320	0.90

* Note that WL = working load.

**Table 5:** Maximum stress in the layer of GFRP cross-arm for broken wire conditions.

Load Designation	Broken Wire Loads (N)			Deformation (mm)	Stress ply (N/mm^2^) First Layer		Compressive Strength (N/mm^2^)	Minimum Factor of Safety (Strength /Stress)
	Vertical	Transverse	Longitudinal		Main	Tie		
1 WL	16,436	8667	25,779	55.70	99.24	92.67	320	3.22
2 WL	32,872	17,334	51,558	109.05	195.60	183.51	320	1.64
3 WL	49,308	26,001	77,337	162.42	291.96	274.35	320	1.10
4 WL	65,744	34,668	103,116	215.79	388.32	365.19	320	0.82

* Note that, WL = working load.

**Table 6:** Summarized minimum factor of safety for normal and broken wire condition.

Load	Deformation (mm)		Minimum Factor of Safety (Strength/Stress)	
	Normal Condition	Broken Wire Condition	Normal Condition	Broken Wire Condition
1 WL	49.60	55.07	5.16	3.22
2 WL	93.77	109.05	2.66	1.64
3 WL	137.96	162.42	1.79	1.10
4 WL	182.15	215.79	1.35	0.82
5 WL	226.35		1.08	
6 WL	270.54		0.90	

* Note that, WL = working load.

**Table 7:** Strength ratio with different loads for GFRP composite cross-arm.

Lamina	Fiber Orientation (°)	1 WL	2 WL	3 WL	4 WL	5 WL	6 WL
lamina 1	0	0.50	0.97	1.44	1.90	2.37	2.84
lamina 2	45	0.30	0.59	0.87	1.16	1.44	1.73
lamina 3	0	0.38	0.73	1.09	1.44	1.79	2.15
lamina 4	−45	0.21	0.40	0.60	0.80	0.99	1.19
lamina 5	0	0.27	0.53	0.79	1.05	1.30	1.56
lamina 6	−45	0.24	0.46	0.68	0.90	1.13	1.35
lamina 7	0	0.37	0.71	1.06	1.40	1.75	2.10
lamina 8	45	0.25	0.49	0.73	0.96	1.20	1.44
lamina 9	0	0.48	0.93	1.37	1.82	2.27	2.72

## Data Availability

All information and data are available within the articles.
